# Artificial Intelligence in Gestational Diabetes Care: A Systematic Review

**DOI:** 10.1177/19322968251355967

**Published:** 2025-08-25

**Authors:** Rawan AlSaad, Ali Elhenidy, Aliya Tabassum, Nour Odeh, Eman AboArqoub, Aya Odeh, Maya AlTamimi, Alaa Abd-alrazaq, Rajat Thomas, Mohammed Bashir, Javaid Sheikh

**Affiliations:** 1 AI Center for Precision Health, Weill Cornell Medicine-Qatar, Doha, Qatar; 2 Computer Engineering and Control Systems Department, Mansoura University, Mansoura, Egypt; 3 Department of Computer Science and Engineering, Qatar University, Doha, Qatar; 4 Jordan University Hospital, Amman, Jordan; 5 Jordanian Royal Medical Services, Amman, Jordan; 6 Qatar Metabolic Institutes, Hamad Medical Corporation, Doha, Qatar

**Keywords:** artificial intelligence, diabetes, gestational diabetes, pregnancy, women’s health

## Abstract

**Background:**

Artificial intelligence (AI) has emerged as a transformative tool for advancing gestational diabetes mellitus (GDM) care, offering dynamic, data-driven methods for early detection, management, and personalized intervention.

**Objective:**

This systematic review aims to comprehensively explore and synthesize the use of AI models in GDM care, including screening, diagnosis, management, and prediction of maternal and neonatal outcomes. Specifically, we examine (1) study designs and population characteristics; (2) the use of AI across different aspects of GDM care; (3) types of input data used for AI modeling; and (4) AI model types, validation strategies, and performance metrics.

**Methods:**

A systematic search was conducted across six electronic databases, identifying 126 eligible studies published up to February 2025. Data extraction and quality appraisal were independently conducted by six reviewers, using a modified version of the Quality Assessment of Diagnostic Accuracy Studies-2 (QUADAS-2) tool for risk of bias assessment.

**Results:**

Among 126 studies, 75% employed retrospective designs, with sample sizes ranging from 17 to over 100 000 participants. Most AI applications (85%) focused on early GDM prediction, while fewer addressed management, outcomes, or monitoring. Classical machine learning dominated (84%), with logistic regression and random forest frequently used. Internal validation was common (68%), but external validation was rare (6%). Our risk of bias appraisal indicated an overall moderate-to-good methodological quality, with notable deficiencies in analysis reporting.

**Conclusions:**

AI demonstrates strong potential to improve GDM prediction, screening, and management. Nonetheless, broader validation, enhanced model interpretability, and prospective studies in diverse populations are needed to translate these technologies into clinical practice.

## Introduction

Gestational diabetes mellitus (GDM) is one of the most common metabolic disorders during pregnancy, affecting approximately 14% to 18% of pregnancies globally, with prevalence rates exhibiting significant regional and population-specific variability due to differences in genetic predisposition, lifestyle factors, and diagnostic criteria.^1-4^ During pregnancy, women with GDM are at increased risk of antenatal complications such as preeclampsia^5^ and higher rates of cesarean delivery, while their offspring are at risk of macrosomia,^6^ neonatal hypoglycemia,^7^ preterm delivery, and admission to neonatal intensive care units. In the long term, mothers with a history of GDM are at elevated risk of developing type 2 diabetes mellitus (T2DM)^8^ and cardiovascular diseases,^9^ and their offspring face an increased risk of childhood obesity.^10^ Given its rising prevalence, particularly in populations with high obesity rates and sedentary lifestyles, there is an urgent need for improved screening, early detection, and personalized management strategies for GDM.

Despite the availability of standardized screening guidelines,^11,12^ traditional approaches for GDM diagnosis and prediction remain suboptimal, time-consuming, and often reactive rather than proactive.^13^ The oral glucose tolerance test (OGTT), which is the gold standard for GDM diagnosis, is invasive, inconvenient, and prone to variability due to factors such as dietary habits, stress, and physiological changes during pregnancy. In addition, risk-based screening methods relying on maternal age, body mass index (BMI), family history, and ethnicity often lack precision, leading to both underdiagnosis and overtreatment. Moreover, disparities in health care access and inconsistent adherence to screening protocols further limit the effectiveness of traditional methods, emphasizing the need for more dynamic, data-driven approaches to improve GDM prediction, early intervention, and personalized management.^12,13^

Artificial intelligence (AI) has emerged as a transformative tool in modern medicine, offering new possibilities for predictive analytics, personalized interventions, and automated decision-making.^14^ In the field of GDM, AI-driven approaches, particularly machine learning (ML) and deep learning (DL) algorithms, have demonstrated promising capabilities in the early risk prediction, continuous glucose monitoring, insulin-dosing recommendations, and outcome prediction. These techniques leverage vast amounts of clinical, biochemical, and lifestyle data to enhance the precision and efficiency of GDM management. The AI applications in GDM range from traditional supervised learning models using structured electronic health records (EHRs) to advanced DL frameworks incorporating real-time sensor data, medical imaging, and genomics. Several recent studies^15-17^ have explored the role of AI in GDM, with models achieving high accuracy in predicting GDM onset based on maternal risk factors, optimizing insulin therapy, and improving continuous glucose monitoring via wearable devices and mobile health (mHealth) applications. In addition, AI-driven decision support systems (DSSs) have been developed to assist clinicians in tailoring treatment plans and providing real-time feedback for dietary and physical activity interventions.

Although a few prior reviews have explored AI applications in GDM care, significant limitations remain.^14-17^ Some reviews, such as the Sub-Saharan Africa-focused review by Gadhia and Loyal,^16^ were geographically restricted and lacked broader applicability beyond low-resource settings. Others, like the review on early GDM prediction using ML,^17^ were limited to models developed before 24 to 28 weeks of pregnancy, overlooking AI applications in diagnosis, management, and outcome prediction later in pregnancy. In addition, previous reviews often focused on specific technologies, such as mobile health (mHealth) platforms,^15^ or were limited to screening and diagnostic models,^14^ without covering the full spectrum of AI approaches or addressing the entire continuum of GDM care. Notably, none of the prior reviews were systematic reviews; they were primarily scoping or narrative reviews, limiting the depth and rigor of their synthesis. In contrast, this systematic review offers a comprehensive synthesis across the entire clinical trajectory of GDM, including screening, diagnosis, management, glycemic control, and prediction of maternal and neonatal outcomes, without restrictions on geographic location, population characteristics, or AI model type. Furthermore, by evaluating methodological quality using a modified Quality Assessment of Diagnostic Accuracy Studies-2 (QUADAS-2) tool, our review critically appraises study rigor, addresses validation gaps, and highlights future research priorities, offering a more holistic and clinically actionable overview than prior work.

This systematic review aims to provide a comprehensive analysis of the current clinical applications of AI in gestational diabetes care, highlighting its potential benefits, limitations, and future directions. The primary research question guiding this review is: *How has artificial intelligence been applied clinically to improve the screening, diagnosis, management, and outcome prediction of gestational diabetes mellitus and its associated maternal and neonatal outcomes during pregnancy and the neonatal period?* By addressing this research question, we aim to provide insights into the current state of AI applications in GDM care. In particular, we synthesize findings across four key aspects: (1) study designs and population characteristics; (2) use of AI in different areas of gestational diabetes care; (3) types of input data used for AI modeling; and (4) AI model types, validation strategies, and performance metrics. This review also assesses methodological strengths and gaps and proposes priorities for future research and clinical implementation.

## Methods

To achieve the objectives of this study, we conducted a systematic review following the Preferred Reporting Items for Systematic Reviews and Meta-Analyses (PRISMA) guidelines.^18^ The corresponding PRISMA checklist is provided in Supplementary Material 1. The following sections present a detailed description of the methods employed in this review.

### Search Strategy

A comprehensive search was conducted on October 4, 2024, across the following electronic databases: MEDLINE (via Ovid), EMBASE (via Ovid), ACM Digital Library, Scopus, IEEE Xplore, and Google Scholar. To ensure ongoing capture of newly published studies, an automated alert was set to run the search query biweekly over a four-month period, concluding on February 4, 2025. Given the extensive volume of results retrieved from Google Scholar, only the first 100 results (equivalent to the first 10 pages) were screened for relevance, in line with common systematic review practices. To identify additional eligible studies, we also performed backward reference list checking (reviewing references cited by included studies) and forward reference list checking (reviewing studies that cited the included studies). The search strategy incorporated two primary categories of keywords: (1) terms related to AI and (2) terms related to Gestational Diabetes. Detailed search queries for each database are provided in Supplementary Material 2.

### Study Eligibility Criteria

The eligibility criteria were designed to ensure the inclusion of studies that specifically evaluated the application of AI for clinical purposes related to GDM during pregnancy, birth, or the neonatal period.

#### Inclusion criteria

- Studies involving pregnant women who were screened or tested for GDM.
- Studies that employed AI algorithms, including ML, DL, or other AI models, for the detection or prediction of GDM or its associated fetal, maternal, and neonatal outcomes.
- Studies addressing outcomes occurring during pregnancy, at birth, or during the neonatal period.
- Original research articles, including retrospective cohort studies, prospective cohort studies, randomized controlled trials, case-control studies, and cross-sectional studies.
- Studies published in English.
- No restrictions on the publication date.

#### Exclusion criteria

- Studies focusing exclusively on outcomes beyond the neonatal period (eg, long-term maternal or child outcomes).
- Review articles, commentaries, editorials, conference abstracts, and preprints.
- Studies not involving the use of AI algorithms for GDM detection, prediction, or management.
- Studies not involving human subjects.

### Study Selection

The study selection process was conducted in three phases. First, duplicate records were removed using EndNote X9 reference management software. In the second phase, the titles and abstracts of the remaining articles were screened for relevance according to the predefined eligibility criteria. In the final phase, the full texts of the shortlisted studies were retrieved and thoroughly assessed for inclusion. The selection process was independently performed by four reviewers. Any disagreements between reviewers were resolved through discussion and consensus; when necessary, a senior reviewer was consulted to reach a final decision.

### Data Extraction

Four reviewers independently extracted data using a standardized Microsoft Excel form. The extracted information was structured to align with the main focus areas of this review. Any disagreements between reviewers were resolved through discussion. The data extraction form was piloted on a sample of ten studies to ensure clarity and consistency and is available in Supplementary Material 3.

### Risk of Bias and Applicability Appraisal

To assess the quality of the studies included in this review, we adapted the QUADAS-2 tool^19^ to better align with the specific objectives of our review. This adaptation involved substituting certain original QUADAS-2 criteria, which were not applicable to our context, with more relevant criteria drawn from the Prediction Model Risk of Bias Assessment Tool (PROBAST).^20^ The modified tool encompassed four key domains tailored to this review: “Participants,” “Index Test” (focused on AI algorithms), “Reference Standard” (representing the ground truth), and “Analysis.”

For each domain, we developed four targeted signaling questions specifically aligned with the aims of this review. In addition to assessing the risk of bias, we also evaluated the applicability of results derived from the first three domains (“Participants,” “Index Test,” and “Reference Standard”). To optimize and calibrate the adapted tool, it was piloted on a subset of ten studies. Subsequently, all included studies were independently evaluated by two reviewers using the finalized modified QUADAS-2 tool (Supplementary Material 4). Any discrepancies between reviewers were discussed and resolved through consensus.

### Data Synthesis

We synthesized the extracted data from the included studies using a narrative approach, providing a comprehensive summary through text, tables, and figures. The synthesis was organized across five key domains: (1) an overview of included studies, summarizing the number of studies, publication years, countries of origin, and publication types; (2) study designs and population details, outlining the types of study designs employed, sample sizes, and participant characteristics; (3) applications of AI in gestational diabetes, detailing the specific clinical goals targeted by AI models, such as screening, diagnosis, risk prediction, and management of GDM; (4) input data used for AI modeling, describing the types and sources of data utilized, including clinical, demographic, laboratory, and imaging data; and (5) AI model types, validation strategies, and performance metrics, summarizing the AI techniques applied, methods used for model validation, and key performance metrics reported.

## Results

### Search Results

**Figure 1.**
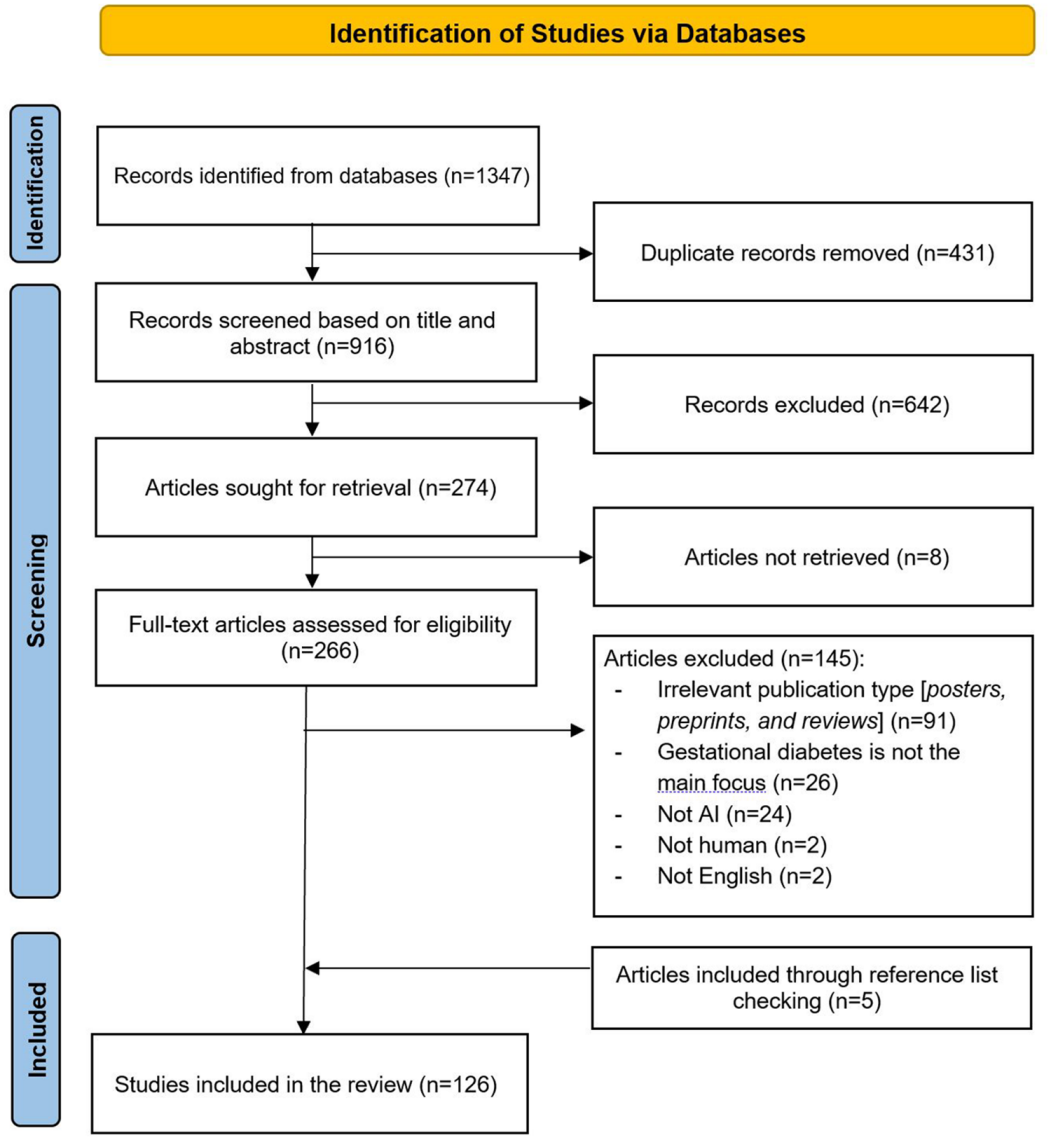
Flowchart of the study selection process.

As illustrated in Figure 1, a total of 1347 records were identified through database searches. After removing 431 duplicate records, 916 records remained for title and abstract screening. Of these, 642 records were excluded for not meeting the inclusion criteria. The full texts of 274 articles were sought for retrieval; however, eight articles could not be retrieved. A total of 266 full-text articles were assessed for eligibility, and 145 were excluded for various reasons, including irrelevant publication type (eg, posters, preprints, and reviews; n = 91), gestational diabetes not being the main focus (n = 26), the study not involving AI (n = 24), not involving human subjects (n = 2), or not being in English (n = 2). In addition, five relevant articles were identified through backward and forward reference list checking. In total, 126 studies were included in the final review.^21-145^

### Overview of Included Studies

**Figure 2.**
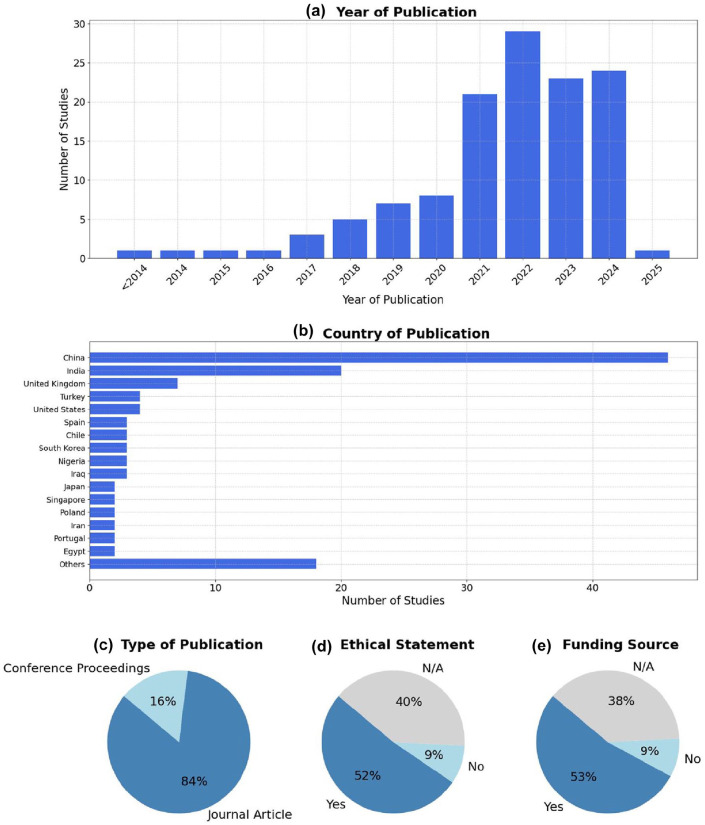
Overview of included studies. (a) Distribution of included studies by year of publication. (b) Country of origin of the included studies. (c) Type of publication. (d) Presence of ethical statements reported in the included studies. (e) Availability of funding sources among the included studies.

As shown in Figure 2 and detailed in Supplemental Table 1, the volume of research on AI applications in GDM has increased substantially in recent years, with over 60% (76/126) of the included studies published between 2021 and 2024. China and India are the leading countries in terms of publication volume, collectively contributing to over 50% (66/126) of all included studies. Geographic representation was broad, with contributions from Asia, Europe, North America, and Africa.

Most studies were journal articles (84%, 106/126), and the remainder were conference papers. Reporting of ethical approval and funding was inconsistent. While 52% (65/126) of the studies reported ethical approval and 53% (67/126) disclosed funding, a considerable proportion did not mention neither, limiting transparency.

### Study Designs and Population Details

Most studies were retrospective in design (75%, 95/126), while only a minority were prospective (21%, 27/126). The majority of studies were retrospective (75%, 95/126), with prospective designs accounting for only 21% (27/126) of the studies. Nearly half of the studies were conducted at a single site (44%, 55/126), 30% (38/126) were multicenter, while 26% (33/126) did not report the number of study sites. Sample sizes varied widely, with a mean of 5468 participants and a range from 17 to over 100 000, indicating a broad spectrum of data set sizes. Where reported, the mean participant age was 32 years, consistent with the typical reproductive age range (Supplemental Table 2).

### Applications of Artificial Intelligence in Gestational Diabetes

**Figure 3.**
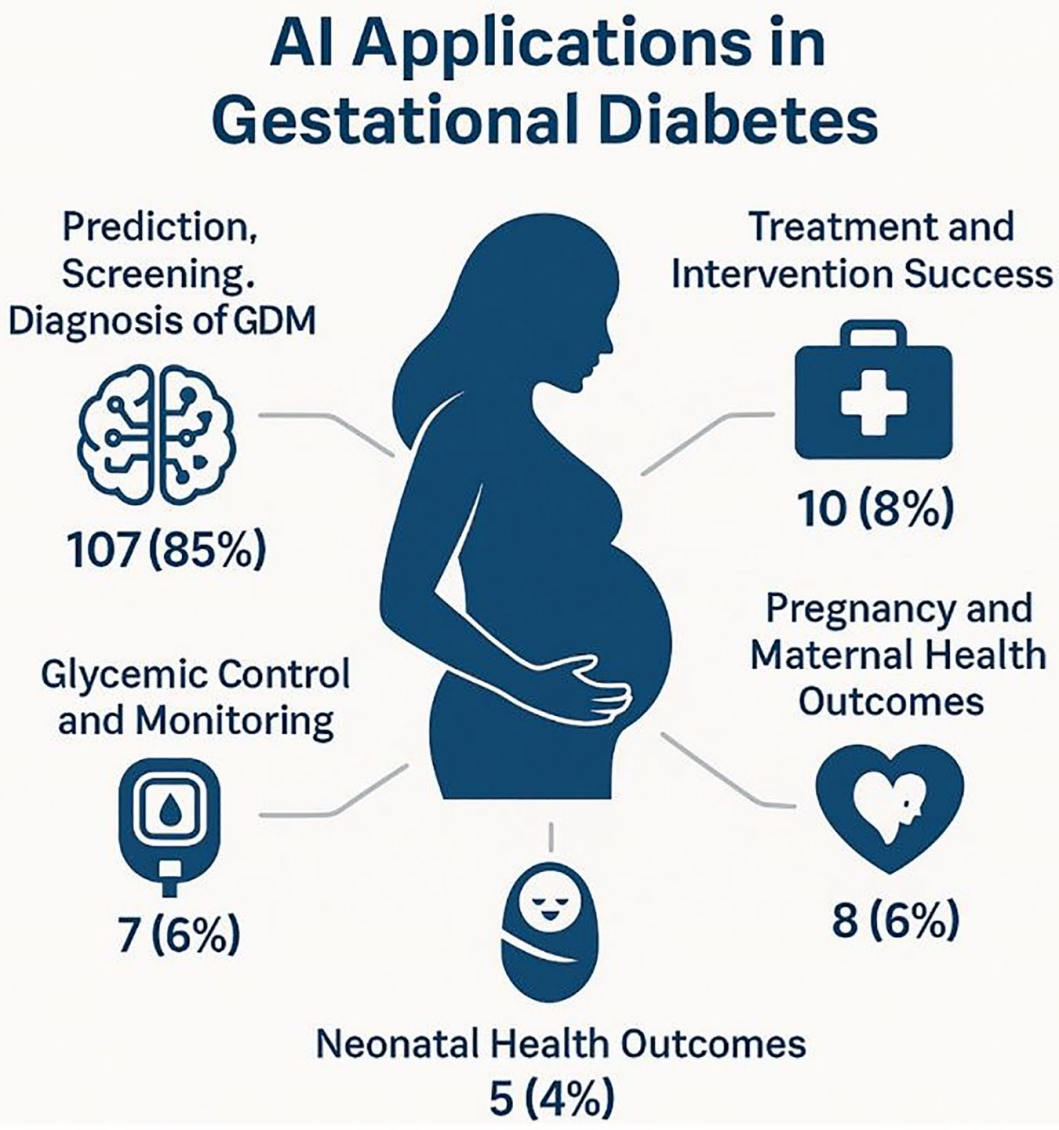
Main applications of artificial intelligence in gestational diabetes care.

Applications of AI in gestational diabetes across the included studies are presented in Supplemental Table 3. As shown in Figure 3, the most common application, observed in 85% (107/126) of studies, was the prediction, screening, and diagnosis of GDM. These studies primarily focused on the early identification of at-risk individuals using clinical, demographic, or biochemical features. A smaller subset, 8% (10/126) of studies, addressed treatment and intervention success, evaluating how AI could predict or optimize responses to therapeutic strategies. Applications targeting pregnancy and maternal health outcomes were reported in 6% (8/126) of studies and focused on predicting pregnancy-related complications in women with GDM, such as preeclampsia, preterm birth, cesarean delivery, and excessive maternal weight gain. Similarly, applications related to glycemic control and monitoring were described in 6% (7/126) of studies, aiming to predict blood glucose fluctuations and support ongoing management of glucose levels throughout pregnancy. Only 4% (5/126) of studies explored AI applications for neonatal health outcomes in pregnancies complicated by GDM, such as predicting birth weight abnormalities or neonatal intensive care unit admission.

At the specific application level, early screening and risk prediction dominated, accounting for 70% (88/126) of studies, underscoring the emphasis on proactive management of GDM. Biomarker-based prediction was reported in 15% (19/126) of studies and referred to the use of specific biological markers, such as blood-based metabolites or genetic data, to improve prediction accuracy. Applications addressing maternal and neonatal health outcomes were identified in 8% (10/126) of studies, while delivery and birth outcome prediction appeared in 6% (8/126) of studies, both focusing on broader pregnancy-related complications associated with GDM. Other niche applications included glucose monitoring and prediction, insulin and pharmacotherapy management, and diet and lifestyle interventions, each comprising 1% (1/126) of studies.

Regarding outcome types, 87% (109/126) of studies focused exclusively on maternal outcomes, 13% (16/126) addressed combined maternal and fetal outcomes, and only 2% (2/126) exclusively targeted fetal outcomes.

### Input Data Used for AI Modeling

**Figure 4.**
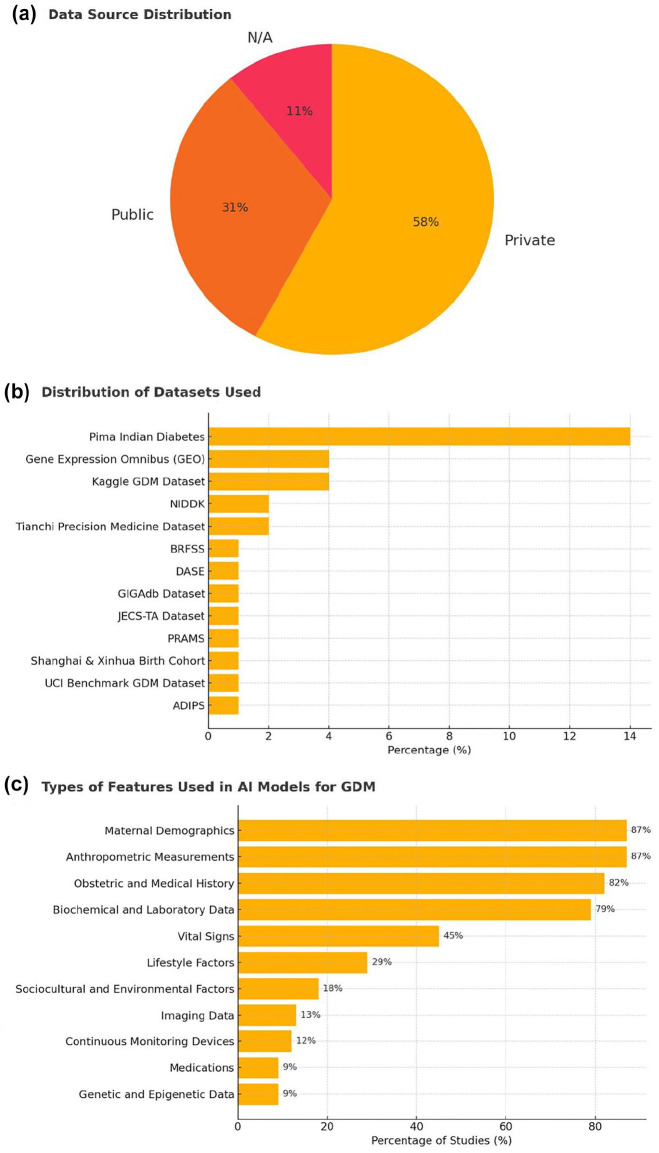
Input data used for AI modeling. (a) Distribution of data sources used across included studies. (b) Distribution of specific data sets utilized for developing AI models for GDM. (c) Types of input features employed in AI models for GDM prediction or diagnosis.

As depicted in Figure 4 and comprehensively outlined in Supplemental Table 4, over half of the studies used private data sets (59%, 74/126), while 32% (39/126) used public data sets. Among the public data sets, the Pima Indian Diabetes data set was the most frequently used (14%, 18/126), followed by GEO (4%, 5/126) and the Kaggle GDM data set (4%, 5/126).

The most commonly used input features were maternal demographics (87%, 110/126), anthropometric measures (87%, 109/126), obstetric history (82%, 103/126), and biochemical or lab data (79%, 99/126). Less frequently used features included vital signs (45%, 57/126), lifestyle factors (29%, 37/126), and genetic data (9%, 11/126). Only a small number of studies incorporated imaging data (13%, 16/126) or continuous monitoring devices (12%, 15/126).

### Artificial Intelligence Model Types, Validation Strategies, and Performance Metrics

**Figure 5.**
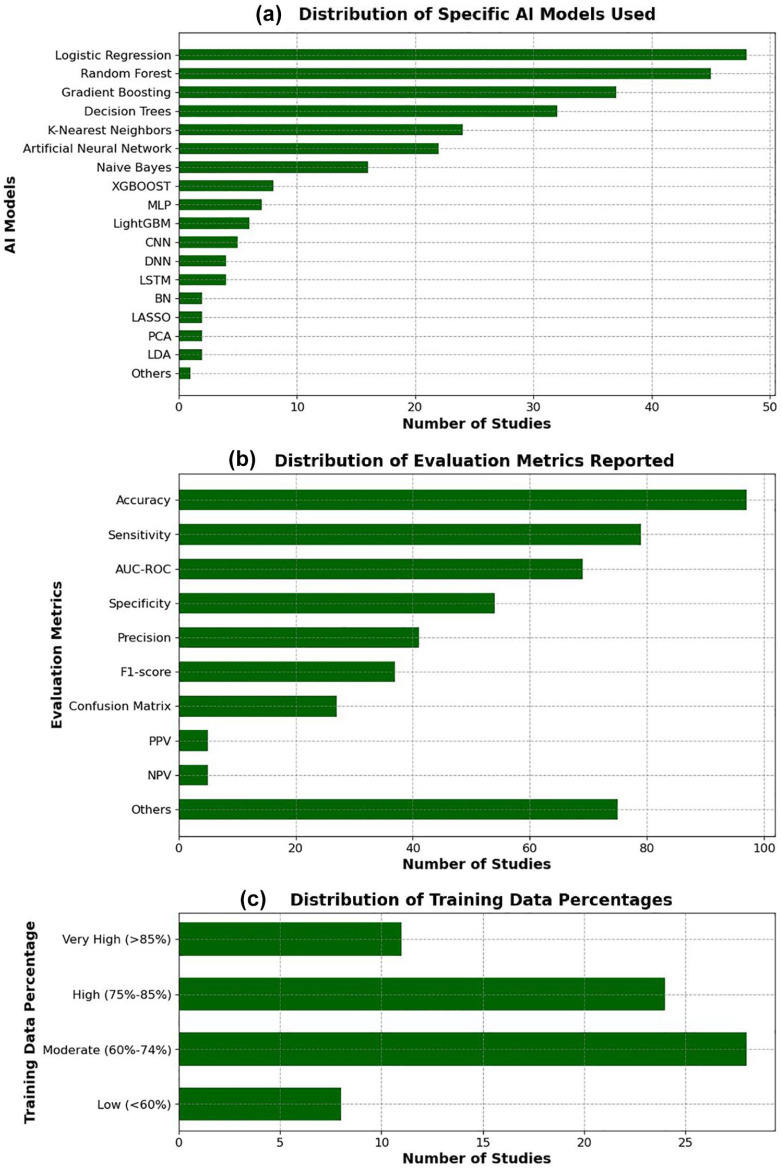
Distribution of AI models, evaluation metrics, and training data percentages among the included studies. (a) Frequency of specific AI models used. (b) Frequency of reported evaluation metrics. (c) Distribution of the proportion of training data used in model development.

**Figure 6.**
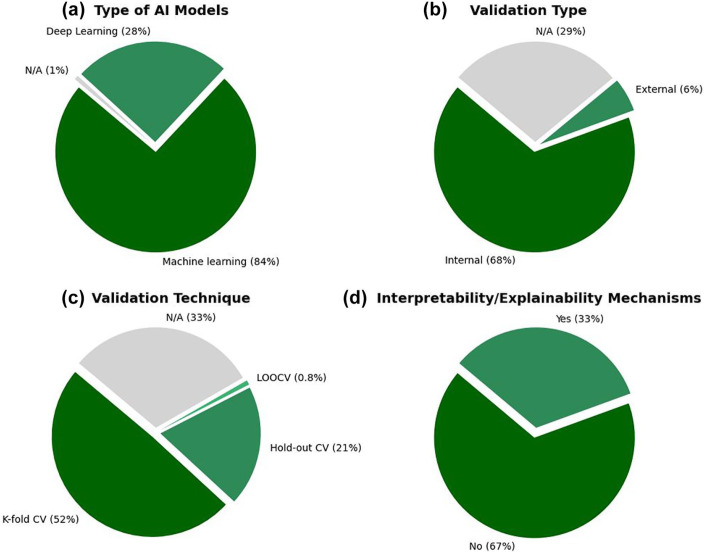
Characteristics of the AI modeling approaches across the included studies. (a) Proportion of machine learning vs deep learning models. (b) Type of validation approach adopted (internal vs external validation). (c) Techniques used for model validation (eg, k-fold cross-validation, hold-out). (d) Implementation of interpretability or explainability mechanisms.

As illustrated in Figures 5 and 6 and comprehensively detailed in Supplemental Table 5, the majority of studies used classical ML models (84%, 106/126), with logistic regression (38%, 48/126), random forest (36%, 45/126), and gradient boosting (29%, 37/126) being the most commonly applied algorithms. Deep learning approaches were used in 28% (36/126) of studies, primarily in more recent publications.

Internal validation was conducted in 68% (86/126) of studies, most commonly through k-fold cross-validation (52%, 66/126). External validation was rare, reported in only 6% (7/126) of studies. Notably, 29% (36/126) of studies did not report the validation method used.

In terms of performance evaluation, the most frequently reported metrics were accuracy (77%, 97/126), sensitivity (63%, 79/126), and area under the receiver operating characteristic curve (AUC-ROC) (55%, 69/126). However, explainability mechanisms were incorporated in only 33% (42/126) of models.

Regarding the proportion of data used for model training, 6% (8/126) of studies used less than 60%, 22% (28/126) used moderate proportions (60%-74%), while 44% (55/126) did not report this information.

### Results of Risk of Bias Appraisal

**Figure 7.**
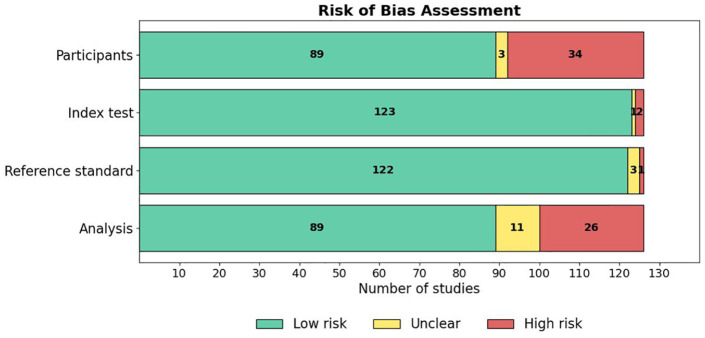
Results of assessment of risk of bias in the studies.

**Figure 8.**
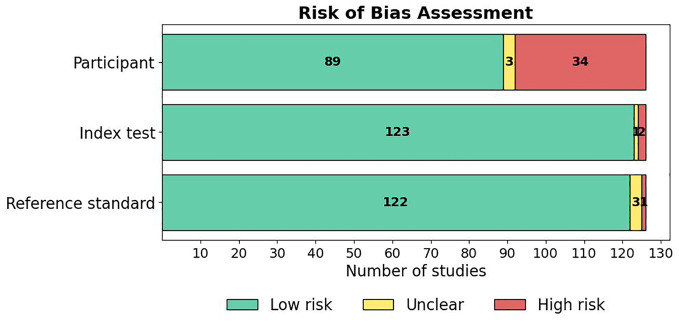
Results of assessment of applicability concerns in the studies.

The risk of bias across the included studies was evaluated using a modified version of the QUADAS-2 tool, with details of the tool and the associated signaling questions provided in Supplementary Material 4. This tool assessed risk of bias across four domains: Participant selection, Index test, Reference standard, and Analysis. A summary of the risk of bias results across studies is presented in Figure 7, and the results of the applicability concerns assessment are shown in Figure 8. A detailed breakdown of the “risk of bias” and “applicability concerns” for each domain in every study is available in Supplementary Material 5. Overall, the majority of studies demonstrated good methodological quality, although certain areas of concern were noted.

#### Participant domain

In the “Participant” domain, 71% (89/126) of studies were assessed as having a low risk of bias (Figure 7). Specifically, 100% (126/126) of studies enrolled a consecutive or random sample of participants (Signaling Question 1.1) and appropriately avoided inappropriate exclusions (1.2). However, only 86% (108/126) of studies included a sufficient sample size (≥100 participants; 1.3), and only 37% (46/126) had a balanced distribution across key subgroups (1.4). Consequently, 27% (34/126) of studies were categorized as high risk, primarily due to concerns regarding sample size and subgroup representation.

#### Index test domain

Nearly all studies described their AI models in detail (Figure 7), with 98% (124/126) providing a comprehensive model description (2.1). Similarly, 99% (125/126) of studies clearly identified all features used in model development (2.2), and 99% (125/126) ensured standardized feature assessment across participants (2.3). In addition, 99% (125/126) of studies collected features without knowledge of the outcome, minimizing the potential for bias (2.4).

#### Reference standard domain

In the “Reference Standard” domain, 97% (122/126) of studies were assessed as low risk (Figure 7). The majority used validated clinical criteria for diagnosing gestational diabetes (3.1) and consistently applied outcome assessments across participants (3.2). Blinding between predictor assessment and outcome determination (3.3) was reported in 99% (125/126) of studies. In addition, nearly all studies (99%, 125/126) minimized the risk of bias related to the timing of predictor and outcome assessments (3.4).

#### Analysis domain

The “Analysis” domain showed slightly more variability (Figure 7). A total of 71% (89/126) of studies were categorized as low risk, whereas 21% (26/126) were assessed as high risk, and 9% (11/126) were unclear. While 77% (97/126) of studies included all participants in the final analysis (4.1), only 50% (63/126) appropriately reported the breakdown of training, validation, and testing sets (4.3), and 67% (84/126) reported model performance appropriately (4.4). Data preprocessing was appropriately described in 98% (124/126) of studies (4.2).

#### Applicability concerns

Regarding applicability, most studies had low concerns and demonstrated strong methodological quality, as shown in Figure 8. Specifically, 71% (89/126) of studies were rated as low concern in the “Participant” domain, 98% (123/126) in the “Index Test” domain, and 97% (122/126) in the “Reference Standard” domain. Only a small proportion of studies were rated as unclear or high concern, primarily due to insufficient reporting of participant characteristics or the use of non-standard reference criteria.

## Discussion

### Principal Findings

This systematic review provides a comprehensive synthesis of the current clinical applications of AI in gestational diabetes care, drawing on 126 original studies. The findings reveal a rapidly evolving landscape, characterized by a surge in research output in recent years: more than 60% of the included studies were published between 2021 and 2024, underscoring the growing recognition of AI’s potential to transform GDM care in response to increasing global disease burden and the pressing need for precision medicine approaches during pregnancy.

The most common application of AI was in the early prediction, screening, and diagnosis of GDM, reported in 85% of the included studies. These models predominantly utilized structured data, such as demographic characteristics, clinical histories, and laboratory results, to enhance the precision and timeliness of GDM detection. This strong focus on early identification reflects a paradigm shift in obstetric care, moving away from reactive diagnosis toward proactive risk stratification and prevention. Early prediction is clinically critical, as it allows for the implementation of lifestyle modifications, glucose monitoring, and pharmacologic interventions that can significantly improve maternal-fetal outcomes. However, the relative underrepresentation of studies targeting later stages of GDM care—such as treatment personalization, glycemic control, and neonatal outcome prediction—highlights a gap in current AI research, particularly considering the complex, dynamic nature of glycemic regulation throughout pregnancy.

In terms of methodological approaches, classical ML algorithms dominated the field, with logistic regression, random forest, and gradient boosting being the most frequently applied. The popularity of these models is understandable given their relatively low computational cost, interpretability, and robust performance on tabular health care data. However, their dominance may also indicate a methodological conservatism, potentially limiting the exploration of more advanced architectures that could exploit richer, multimodal data sources. Deep learning techniques, although applied in only 28% of studies, are gradually gaining traction, particularly in projects leveraging imaging, continuous glucose monitoring, or omics data. This gradual adoption suggests an encouraging trend toward more sophisticated modeling strategies, aligned with the increasing availability of high-dimensional and longitudinal data sets in maternal-fetal medicine.

Several important methodological challenges persist. Internal validation, predominantly via k-fold cross-validation, was reported in the majority of studies (68%), reflecting efforts to reduce overfitting. However, external validation—crucial for assessing model generalizability across populations and settings—was exceedingly rare (6%). The absence of external validation remains a critical barrier to clinical translation, as AI models trained and validated on homogeneous or geographically restricted data sets may fail to perform adequately in more diverse real-world populations. Furthermore, nearly one-third of studies did not adequately describe their validation strategies, undermining reproducibility and raising concerns about potential reporting biases that could inflate model performance estimates.

The overreliance on accuracy as a primary performance metric, particularly in imbalanced data sets where GDM prevalence may be low, can lead to misleading assessments of clinical utility. Incorporating complementary metrics such as sensitivity, specificity, and F1-score would provide a more nuanced and clinically relevant understanding of model strengths and weaknesses. Moreover, the explainability of AI models remains underdeveloped; only one-third of studies incorporated any form of interpretability mechanisms. In a high-stakes context like pregnancy care, where clinical decisions directly impact maternal and neonatal health, black-box models are unlikely to gain clinician trust or regulatory approval.

Our risk of bias appraisal indicated overall moderate-to-good methodological quality, with notable strengths observed in the index test and reference standard used. Nevertheless, deficiencies in analysis reporting, especially regarding the documentation of training and testing set partitions, were notable. Poor reporting not only compromises study reproducibility but also hampers the ability to critically appraise the robustness of model development pipelines.

### Research and Practical Implications

The findings of this review have several important implications for the future development and clinical translation of AI applications in gestational diabetes care. First, while the focus on early prediction and diagnosis is justified by the potential for preventive interventions, there is a clear need to broaden the scope of AI research to encompass the full continuum of gestational diabetes care. Future studies should explore the use of AI for treatment optimization, dynamic glucose monitoring, individualized insulin dosing, and prediction of maternal and neonatal outcomes beyond the diagnosis of GDM itself. Such expansion is critical for realizing AI’s potential not only as a diagnostic aid but also as a tool for comprehensive pregnancy management and postnatal care.

Second, the predominance of studies relying on retrospective data and single-center cohorts highlights the urgent need for prospective, multicenter studies with diverse populations. The AI models trained and validated on narrow, homogeneous data sets risk poor generalizability and may exacerbate existing disparities in GDM care if deployed without external validation. Future research should prioritize the development of models using multiethnic, geographically diverse cohorts and should report performance across clinically meaningful subgroups, such as by ethnicity, BMI category, or age. This will be essential for ensuring equitable AI adoption in obstetric practice.

Third, enhancing model transparency and interpretability must become a central research priority. The low proportion of studies incorporating explainability techniques is concerning, given that clinical decision-making in pregnancy demands not only accuracy but also clinician understanding and justification of model outputs. Researchers should routinely integrate explainable AI (XAI) methods, such as SHapley Additive exPlanations (SHAP) values, Local Interpretable Model-agnostic Explanations (LIME), attention maps, or counterfactual explanations, and report how these mechanisms can inform clinical actionability. The development of interpretable-by-design models, rather than post hoc explanations, may be particularly valuable for sensitive clinical domains such as maternal-fetal medicine.

Fourth, to facilitate reproducibility, critical appraisal, and eventual clinical translation, future studies must adhere to standardized reporting guidelines such as transparent reporting of a multivariable prediction model for individual prognosis Or diagnosis–artificial intelligence (TRIPOD-AI) and Consolidated Standards of Reporting Trials–Artificial Intelligence (CONSORT-AI).^146^ Transparent documentation of data sources, preprocessing steps, model development pipelines, training-validation-testing splits, and full performance metrics should be considered mandatory.

Finally, collaboration between AI developers, obstetricians, endocrinologists, data scientists, and patients will be vital. Co-designing AI tools with end-users in mind can improve clinical relevance, usability, and trust. Moreover, pilot implementation studies and randomized trials evaluating the clinical impact of AI-guided interventions in GDM care are urgently needed. Without such evidence, the clinical deployment of AI will remain aspirational rather than transformational.

### Limitations

This review has several limitations that should be considered when interpreting the findings. First, it included only studies published in English, which may have resulted in the exclusion of relevant research conducted in other languages. Second, although this review systematically synthesized a large number of studies, it did not conduct a meta-analytical synthesis of model performance. The decision against meta-analysis was primarily due to the substantial heterogeneity across studies in terms of study designs, AI model types, data sources, feature sets, validation strategies, and performance reporting. Studies varied widely in their input features (eg, clinical vs imaging data), prediction targets (eg, GDM onset vs maternal or neonatal complications), and evaluation metrics (eg, accuracy, AUC-ROC, sensitivity, specificity), often without standardized thresholds or consistent methodologies. In addition, many studies lacked sufficient statistical reporting (eg, confidence intervals, calibration plots), making it inappropriate and potentially misleading to pool performance estimates quantitatively. Therefore, while a meta-analysis would have been valuable for producing pooled estimates of AI model performance, the methodological diversity and incomplete reporting across studies precluded a meaningful aggregation of results in this review. Future systematic reviews with meta-analysis are recommended, particularly when sufficient homogeneous studies become available, to quantitatively compare the predictive accuracy of AI models across different application domains, data modalities, and model complexities. Finally, while this review comprehensively evaluated the clinical applications of AI during pregnancy and the neonatal period, it excluded studies that focused solely on long-term maternal or offspring outcomes beyond the immediate postpartum period. Given the increasing interest in the long-term metabolic and cardiovascular risks associated with GDM exposure, future research should extend the scope to assess how AI can support prediction and prevention strategies beyond birth.

## Conclusions

This systematic review provides the most comprehensive synthesis to date of clinical applications of AI in GDM. Analyzing 126 studies, we observed a rapid growth in research efforts, reflecting increasing recognition of AI’s potential to transform GDM care. The majority of studies focused on early prediction, screening, and diagnosis using structured clinical data, with fewer addressing treatment personalization, glycemic control, or neonatal outcomes. Methodologically, classical ML models dominated, while DL approaches are emerging as the field matures. Despite encouraging model performances, critical challenges remain, including limited external validation, underreporting of methodological details, and insufficient integration of interpretability mechanisms.

Addressing these gaps will be crucial to advancing AI from theoretical promise to clinical utility. Future research must prioritize prospective, multicenter studies, model transparency, standardized reporting, and collaboration with clinical end-users. Moreover, expanding AI applications to support the full continuum of GDM care—from early risk prediction to postpartum follow-up—will be essential for maximizing patient benefit. Overall, while AI holds strong promise for enhancing the management of GDM, realizing its clinical impact will require a more rigorous, transparent, and patient-centered research agenda.

## Supplemental Material

sj-docx-1-dst-10.1177_19322968251355967 – Supplemental material for Artificial Intelligence in Gestational Diabetes Care: A Systematic Review Supplemental material, sj-docx-1-dst-10.1177_19322968251355967 for Artificial Intelligence in Gestational Diabetes Care: A Systematic Review by Rawan AlSaad, Ali Elhenidy, Aliya Tabassum, Nour Odeh, Eman AboArqoub, Aya Odeh, Maya AlTamimi, Alaa Abd-alrazaq, Rajat Thomas, Mohammed Bashir and Javaid Sheikh in Journal of Diabetes Science and Technology

sj-docx-2-dst-10.1177_19322968251355967 – Supplemental material for Artificial Intelligence in Gestational Diabetes Care: A Systematic Review Supplemental material, sj-docx-2-dst-10.1177_19322968251355967 for Artificial Intelligence in Gestational Diabetes Care: A Systematic Review by Rawan AlSaad, Ali Elhenidy, Aliya Tabassum, Nour Odeh, Eman AboArqoub, Aya Odeh, Maya AlTamimi, Alaa Abd-alrazaq, Rajat Thomas, Mohammed Bashir and Javaid Sheikh in Journal of Diabetes Science and Technology

sj-docx-3-dst-10.1177_19322968251355967 – Supplemental material for Artificial Intelligence in Gestational Diabetes Care: A Systematic Review Supplemental material, sj-docx-3-dst-10.1177_19322968251355967 for Artificial Intelligence in Gestational Diabetes Care: A Systematic Review by Rawan AlSaad, Ali Elhenidy, Aliya Tabassum, Nour Odeh, Eman AboArqoub, Aya Odeh, Maya AlTamimi, Alaa Abd-alrazaq, Rajat Thomas, Mohammed Bashir and Javaid Sheikh in Journal of Diabetes Science and Technology

sj-docx-4-dst-10.1177_19322968251355967 – Supplemental material for Artificial Intelligence in Gestational Diabetes Care: A Systematic Review Supplemental material, sj-docx-4-dst-10.1177_19322968251355967 for Artificial Intelligence in Gestational Diabetes Care: A Systematic Review by Rawan AlSaad, Ali Elhenidy, Aliya Tabassum, Nour Odeh, Eman AboArqoub, Aya Odeh, Maya AlTamimi, Alaa Abd-alrazaq, Rajat Thomas, Mohammed Bashir and Javaid Sheikh in Journal of Diabetes Science and Technology

sj-docx-5-dst-10.1177_19322968251355967 – Supplemental material for Artificial Intelligence in Gestational Diabetes Care: A Systematic Review Supplemental material, sj-docx-5-dst-10.1177_19322968251355967 for Artificial Intelligence in Gestational Diabetes Care: A Systematic Review by Rawan AlSaad, Ali Elhenidy, Aliya Tabassum, Nour Odeh, Eman AboArqoub, Aya Odeh, Maya AlTamimi, Alaa Abd-alrazaq, Rajat Thomas, Mohammed Bashir and Javaid Sheikh in Journal of Diabetes Science and Technology
