# Supplementary material for: Artificial Intelligence in Gestational Diabetes Care: A Systematic Review
Source: J Diabetes Sci Technol. 2025 Aug 25:19322968251355967. Online ahead of print. doi: 10.1177/19322968251355967 (PMC12380749; doi:10.1177/19322968251355967)
Supplement: sj-docx-2-dst-10.1177_19322968251355967 – Supplemental material for Artificial Intelligence in Gestational Diabetes Care: A Systematic Review [file sj-docx-2-dst-10.1177_19322968251355967.docx]

**Multimedia Appendix 2: Search Strategy**

Database(s): **Ovid MEDLINE(R) ALL**1946 to October 04, 2024
Search Strategy:

| **#** | **Searches** | **Results** |
| --- | --- | --- |
| 1 | exp Artificial Intelligence/ | 210458 |
| 2 | "Artificial Intelligence".tw. | 49243 |
| 3 | exp Machine Learning/ | 76819 |
| 4 | "Machine Learning".tw. | 110381 |
| 5 | exp Deep Learning/ | 23016 |
| 6 | "Deep Learning".tw. | 62042 |
| 7 | "Decision Tree*".tw. | 17350 |
| 8 | "Support Vector Machine*".tw. | 29342 |
| 9 | "Recurrent Neural Network*".tw. | 4814 |
| 10 | "Convolutional Neural Network*".tw. | 29060 |
| 11 | "Artificial Neural Network*".tw. | 19320 |
| 12 | "Deep Neural Network*".tw. | 11185 |
| 13 | "Random Forest*".tw. | 29198 |
| 14 | "Long Short-Term Memory*".tw. | 5754 |
| 15 | "Autoencoder".tw. | 3243 |
| 16 | "Gradient Boost*".tw. | 8111 |
| 17 | "Multilayer Perceptron".tw. | 3198 |
| 18 | "Ensemble learning".tw. | 2318 |
| 19 | "Generative Pre-trained Transformer*".tw. | 498 |
| 20 | "Large Language Model*".tw. | 2777 |
| 21 | "Generative AI".tw. | 590 |
| 22 | "transformer model*".tw. | 849 |
| 23 | Gestation*.tw. | 261918 |
| 24 | pregnan*.tw. | 625988 |
| 25 | exp Diabetes, Gestational/ | 19145 |
| 26 | diabet*.tw. | 826805 |
| 27 | 1 or 2 or 3 or 4 or 5 or 6 or 7 or 8 or 9 or 10 or 11 or 12 or 13 or 14 or 15 or 16 or 17 or 18 or 19 or 20 or 21 or 22 | 364042 |
| 28 | 23 or 24 | 747032 |
| 29 | 25 or 26 | 829081 |
| 30 | 27 and 28 and 29 | 299 |
| 31 | limit 30 to (english language and humans) | 222 |

Database(s): **Embase**1974 to 2024 Week 40
Search Strategy:

| **#** | **Searches** | **Results** |
| --- | --- | --- |
| 1 | exp Artificial Intelligence/ | 116657 |
| 2 | "Artificial Intelligence".tw. | 58108 |
| 3 | exp Machine Learning/ | 516456 |
| 4 | "Machine Learning".tw. | 129246 |
| 5 | exp Deep Learning/ | 65030 |
| 6 | "Deep Learning".tw. | 71443 |
| 7 | "Decision Tree*".tw. | 23901 |
| 8 | "Support Vector Machine*".tw. | 34706 |
| 9 | "Recurrent Neural Network*".tw. | 5402 |
| 10 | "Convolutional Neural Network*".tw. | 33855 |
| 11 | "Artificial Neural Network*".tw. | 22156 |
| 12 | "Deep Neural Network*".tw. | 12457 |
| 13 | "Random Forest*".tw. | 35309 |
| 14 | "Long Short-Term Memory*".tw. | 5750 |
| 15 | "Autoencoder".tw. | 3582 |
| 16 | "Gradient Boost*".tw. | 9549 |
| 17 | "Multilayer Perceptron".tw. | 3515 |
| 18 | "Ensemble learning".tw. | 2569 |
| 19 | "Generative Pre-trained Transformer*".tw. | 443 |
| 20 | "Large Language Model*".tw. | 2917 |
| 21 | "Generative AI".tw. | 598 |
| 22 | "transformer model*".tw. | 887 |
| 23 | Gestation*.tw. | 369268 |
| 24 | pregnan*.tw. | 814090 |
| 25 | exp Diabetes, Gestational/ | 15944 |
| 26 | diabet*.tw. | 1257712 |
| 27 | 1 or 2 or 3 or 4 or 5 or 6 or 7 or 8 or 9 or 10 or 11 or 12 or 13 or 14 or 15 or 16 or 17 or 18 or 19 or 20 or 21 or 22 | 617216 |
| 28 | 23 or 24 | 975056 |
| 29 | 25 or 26 | 1263771 |
| 30 | 27 and 28 and 29 | 593 |
| 31 | limit 30 to (english language and humans) | 559 |
| 32 | limit 31 to "remove medline records" | 251 |

Database(s): **CINAHL**

Mon, October 7, 2024 12:42:32 PM

| **#** | **Query** | **Results** |
| --- | --- | --- |
| S1 | MH "Artificial Intelligence" | 14,021 |
| S2 | AB "Artificial Intelligence" | 5,969 |
| S3 | MW "Machine Learning" | 7,228 |
| S4 | AB "Machine Learning" | 10,655 |
| S5 | MW "Deep Learning" | 2,954 |
| S6 | AB "Deep Learning" | 4,264 |
| S7 | AB "Decision Tree*" | 3,315 |
| S8 | AB "Support Vector Machine*" | 2,932 |
| S9 | AB "Recurrent Neural Network*" | 240 |
| S10 | AB "Convolutional Neural Network*" | 1,969 |
| S11 | AB "Artificial Neural Network*" | 1,316 |
| S12 | AB "Deep Neural Network*" | 508 |
| S13 | AB "Random Forest*" | 3,287 |
| S14 | AB "Long Short-Term Memory*" | 313 |
| S15 | AB "Autoencoder" | 89 |
| S16 | AB "Gradient Boost*" | 925 |
| S17 | AB "Multilayer Perceptron" | 252 |
| S18 | AB "Ensemble learning" | 152 |
| S19 | AB "Generative Pre-trained Transformer*" | 42 |
| S20 | AB "Large Language Model*" | 206 |
| S21 | AB "Generative AI" | 68 |
| S22 | AB "transformer model*" | 37 |
| S23 | AB Gestation* | 61,207 |
| S24 | AB pregnan* | 128,450 |
| S25 | MW Gestational diabetes | 9,496 |
| S26 | AB diabet* | 170,613 |
| S27 | S1 OR S2 OR S3 OR S4 OR S5 OR S6 OR S7 OR S8 OR S9 OR S10 OR S11 OR S12 OR S13 OR S14 OR S15 OR S16 OR S17 OR S18 OR S19 OR S20 OR S21 OR S22 | 36,815 |
| S28 | S23 OR S24 | 155,238 |
| S29 | S25 OR S26 | 173,967 |
| S30 | S27 AND S28 AND S29 | 60 |
| S31 | S30 Narrow by Language: - english | 60 |

Database(s): **APA PsychInfo**

Mon, October 7, 2024 12:56:31 PM

| **#** | **Query** | **Results** |
| --- | --- | --- |
| S1 | MH "Artificial Intelligence" | 39,957 |
| S2 | AB "Artificial Intelligence" | 7,774 |
| S3 | MW "Machine Learning" | 31,073 |
| S4 | AB "Machine Learning" | 11,950 |
| S5 | MW "Deep Learning" | 15,922 |
| S6 | AB "Deep Learning" | 3,548 |
| S7 | AB "Decision Tree*" | 2,323 |
| S8 | AB "Support Vector Machine*" | 3,594 |
| S9 | AB "Recurrent Neural Network*" | 1,031 |
| S10 | AB "Convolutional Neural Network*" | 1,674 |
| S11 | AB "Artificial Neural Network*" | 2,185 |
| S12 | AB "Deep Neural Network*" | 1,055 |
| S13 | AB "Random Forest*" | 1,986 |
| S14 | AB "Long Short-Term Memory*" | 494 |
| S15 | AB "Autoencoder" | 249 |
| S16 | AB "Gradient Boost*" | 402 |
| S17 | AB "Multilayer Perceptron" | 276 |
| S18 | AB "Ensemble learning" | 219 |
| S19 | AB "Generative Pre-trained Transformer*" | 27 |
| S20 | AB "Large Language Model*" | 229 |
| S21 | AB "Generative AI" | 99 |
| S22 | AB "transformer model*" | 41 |
| S23 | AB Gestation* | 15,915 |
| S24 | AB pregnan* | 55,784 |
| S25 | MW Gestational diabetes | 1,043 |
| S26 | AB diabet* | 37,844 |
| S27 | S1 OR S2 OR S3 OR S4 OR S5 OR S6 OR S7 OR S8 OR S9 OR S10 OR S11 OR S12 OR S13 OR S14 OR S15 OR S16 OR S17 OR S18 OR S19 OR S20 OR S21 OR S22 | 30,851 |
| S28 | S23 OR S24 | 64,301 |
| S29 | S25 OR S26 | 37,891 |
| S30 | S27 AND S28 AND S29 | 9 |
| S31 | S30 Narrow by Language: - english | 9 |

| **Database** | **Search query** | **Hits** |
| --- | --- | --- |
| **Scopus** | ( TITLE-ABS-KEY ( "Artificial Intelligence" OR "Machine Learning" OR "Deep Learning" OR "Decision Tree*" OR "Support Vector Machine*" OR "Recurrent Neural Network*" OR "Convolutional Neural Network*" OR "Artificial Neural Network*" OR "Deep Neural Network*" OR "Random Forest*" OR "Long Short-Term Memory*" OR "Autoencoder" OR "Gradient Boost*" OR "Multilayer Perceptron" OR "Ensemble learning" OR "Generative Pre-trained Transformer*" OR "Large Language Model*" OR "Generative AI" OR "transformer model*" ) AND TITLE-ABS-KEY ( gestation* OR pregnan* ) AND TITLE-ABS-KEY ( diabet* ) ) AND ( LIMIT-TO ( DOCTYPE , "ar" ) OR LIMIT-TO ( DOCTYPE , "cp" ) ) | 640 |
| **IEEE Xplore** | ("Abstract":"Artificial Intelligence" OR "Abstract":"Machine Learning" OR "Abstract":"Deep Learning" OR "Abstract":"Decision Tree" OR "Abstract":"Support Vector Machine*" OR "Abstract":"Recurrent Neural Network*" OR "Abstract":"Convolutional Neural Network*" OR "Abstract":"Artificial Neural Network*" OR "Abstract":"Deep Neural Network*" OR "Abstract":"Random Forest" OR "Abstract":"Long Short-Term Memory" OR "Abstract":"Autoencoder" OR "Abstract":"Gradient Boost" OR "Abstract":"Multilayer Perceptron" OR "Abstract":"Ensemble learning" OR "Abstract":"Generative Pre-trained Transformer*" OR "Abstract":"Large Language Model*" OR "Abstract":"Generative AI" OR "Abstract":"transformer model*") AND ("Abstract":Gestation* OR "Abstract":pregnan*) AND ("Abstract":diabet* ) | 61 |
| **ACM digital Library** | [[Abstract: "artificial intelligence"] OR [Abstract: "machine learning"] OR [Abstract: "deep learning"] OR [Abstract: "decision tree*"] OR [Abstract: "support vector machine*"] OR [Abstract: "recurrent neural network*"] OR [Abstract: "convolutional neural network*"] OR [Abstract: "artificial neural network*"] OR [Abstract: "deep neural network*"] OR [Abstract: "random forest*"] OR [Abstract: "long short-term memory*"] OR [Abstract: "autoencoder"] OR [Abstract: "gradient boost*"] OR [Abstract: "multilayer perceptron"] OR [Abstract: "ensemble learning"] OR [Abstract: "generative pre-trained transformer*"] OR [Abstract: "large language model*"] OR [Abstract: "generative ai"] OR [Abstract: "transformer model*"]] AND [[Abstract: gestation*] OR [Abstract: pregnan*]] AND [Abstract: diabet*] | 4 |
| **Google Scholar** | ("Artificial Intelligence" OR "Machine Learning" OR "Deep Learning" OR "Convolutional Neural Network*" OR "Generative Pre-trained Transformer*" OR "Large Language Model*" OR "Generative AI" OR "transformer model*") AND (Gestation* OR pregnan*) AND diabet* | 100 |
