# Supplementary material for: Artificial Intelligence in Gestational Diabetes Care: A Systematic Review
Source: J Diabetes Sci Technol. 2025 Aug 25:19322968251355967. Online ahead of print. doi: 10.1177/19322968251355967 (PMC12380749; doi:10.1177/19322968251355967)
Supplement: sj-docx-3-dst-10.1177_19322968251355967 – Supplemental material for Artificial Intelligence in Gestational Diabetes Care: A Systematic Review [file sj-docx-3-dst-10.1177_19322968251355967.docx]

**Multimedia Appendix 3: Data Extraction Form**

| **Extracted data** | **Definition** |
| --- | --- |
| **1. Study Characteristics** | |
| 1.1 Author | The first author of the study. |
| 1.2 Year of publication | The year in which the study was published. |
| 1.3 Type of publication | The venue where the study was published: peer-reviewed journal, book chapters, dissertations, or conference proceedings. |
| 1.4 Country of publication | The country where the study was published. |
| 1.5 Ethical statement | Indicate whether the study explicitly includes an ethical statement addressing approval for the use of data and compliance with ethical standards:   - **Yes**: The study uses closed dataset and mentions ethical approval from an institutional review board (IRB), ethics committee, or similar authority. - **No**: The study uses closed dataset and does not include an ethical statement or mention ethical approval. - **N/A**: The study uses public dataset. |
| 1.6 Funding source | Indicate whether the study mentions a funding source and provide details as applicable:   - **Yes**: The study declares a specific funding source. - **No**: The study explicitly states that no funding was received for the work. - **N/A**: The study does not mention funding or its absence. |
| **2. Study Design** | |
| 2.1 Research design | The research design or methodology employed in a study, which can be categorized as:   - Retrospective - Prospective - Retrospective & Prospective |
| 2.2 Number of sites | Indicate the total number of clinics or hospitals involved in the study |
| 2.3 Number of participants | Report the total original number of participants included in the study. Don't worry if some participants are excluded during preprocessing. |
| 2.4 Mean age of participants | State the average age of participants (if available). |
| 2.5 Age range | Provide the minimum and maximum ages of participants in the study (if available). Example: 18-45. |
| **3. Applications** | |
| 3.1 Main application | 1. **Prediction, screening, diagnosis of GDM**  - Was the AI model designed to **predict** the onset or risk of gestational diabetes? - Was the AI model designed to **detect** or **confirm** gestational diabetes. - If yes, fill in next column (3.3 Stage of pregnancy): At which stage/trimester of pregnancy was the prediction made (e.g., pre-pregnancy, first trimester, second trimester)?   *Examples*:   - Early screening models for gestational diabetes based on maternal characteristics. - Prediction using biomarkers like HbA1c, fasting glucose, or insulin resistance. - Predictive tools integrating genetic and epigenetic data.  1. **Pregnancy and Maternal Health Outcomes**   Prediction or risk assessment of maternal and pregnancy-related health outcomes influenced by gestational diabetes.   - *Maternal Complications:*   - Risk of **preeclampsia** or gestational hypertension.   - Likelihood of **preterm birth** or early labor induction.   - **Mode of delivery (vaginal/** **cesarean**)   - Predictions related to **maternal weight gain and obesity management** during pregnancy. - *Fetal and Pregnancy-Related Complications:*   - **Fetal growth complications:**     - Macrosomia (large-for-gestational-age, LGA).     - Small-for-gestational-age (SGA).   - Risks associated with **shoulder dystocia** or birth trauma.   - Stillbirth or other serious pregnancy outcomes associated with gestational diabetes.  1. **Neonatal Health Outcomes**   Assessment of neonatal health influenced by gestational diabetes.  *Examples*:   - **Short-term neonatal outcomes:**   - Apgar scores.   - NICU admission rates.   - Neonatal hypoglycemia and its management.   - Respiratory distress or transient tachypnea of the newborn (TTN). - **Long-term outcomes:**   - Developmental delays.   - Risk of childhood obesity or type 2 diabetes in offspring.  1. **Glycemic Control and Monitoring**   Effectiveness of AI in predicting, managing, or monitoring maternal glucose levels.  *Examples*:   - Prediction of **hyperglycemia** or **hypoglycemia episodes.** - Personalized glycemic monitoring through continuous glucose monitoring (CGM) devices. - Adaptive insulin therapy or oral medication recommendations.  1. **Treatment and Intervention Success**   Evaluation of the effectiveness of treatments or interventions for managing gestational diabetes.  Examples:   - AI models optimizing medical treatments:   - **Insulin** therapy dosage prediction and timing.   - Efficacy of metformin or other pharmacological treatments. - AI-guided lifestyle interventions:   - Diet and meal planning.   - Exercise regimens tailored for glucose management.   - Monitoring patient adherence to treatments or recommendations. |
| 3.2 Specific Applications | Indicate the specific applications, stick to examples listed here and separate them by comma:   - 1. **Early Screening & Risk Prediction** – Identifying individuals at risk of GDM.   2. **Biomarker-Based Prediction** – Using biomarkers (lipid, maternal, placental, etc.) for diagnosis or monitoring.   3. **Glucose Monitoring & Prediction**– Predicting glucose levels and hyperglycemia episodes.   4. **Insulin & Pharmacotherapy Management** – Adaptive insulin therapy and predicting the need for pharmacotherapy.   5. **Diet & Lifestyle Interventions** – AI-driven diet planning, exercise regimens, and adherence monitoring.  1. **Maternal & Neonatal Health Outcomes** – Predicting adverse pregnancy outcomes related to GDM (Macrosomia, large for gestational age LGA) 2. **Delivery & Birth Outcome Prediction** – Mode of delivery, birth weight, Apgar scores, NICU admission, etc. |
| 3.3 Stage of pregnancy | **For Main Application 1 studies only**: if the AI model was designed to predict the onset of gestational diabetes, indicate at which stage of pregnancy was prediction made (Pre-pregnancy, T1: first trimester, T2: second trimester, T3: third trimester). |
| 3.4 Outcome type | - **Mother**: Includes outcomes related specifically to maternal health, such as gestational hypertension, preeclampsia, mode of delivery, or maternal weight management. - **Fetus**: Covers outcomes directly affecting the fetus, such as fetal growth (e.g., macrosomia, small-for-gestational-age), or stillbirth. - **Both**: Encompasses outcomes that involve both maternal and fetal health, such as pregnancy complications affecting both mother and baby (e.g., preterm birth risk, neonatal health influenced by maternal glucose control). |
| **4. Data Characteristics** | |
| 4.1 Data source | The source of data that was used for developing the algorithms.   - **Open datasets**: publicly available datasets that anyone can access, use, and share, often free of charge (e.g. Kaggle, PIMA Indian dataset). - **Closed datasets:** proprietary datasets with restricted access, often controlled by licensing agreements or other legal constraints. - **Open & closed** |
| 4.2 Dataset name | If the data used is an open (public) dataset, indicate the dataset name (e.g. PIMA India dataset). |
| 4.3 Category of Data input to AI algorithm | Types of data used as input for the algorithm:  **1. Maternal Demographics**: Age, Ethnicity/Race, Socioeconomic Status, Family History of Diabetes.  **2. Anthropometric Measurements:** Body Mass Index (BMI), Pre-pregnancy Weight, Height, Weight Gain During Pregnancy.  **3. Obstetric and Medical History**: Parity (number of pregnancies), History of Gestational Diabetes Mellitus (GDM), History of Macrosomia (birth weight >4 kg), Pre-existing Hypertension, Pre-existing Type 1 or Type 2 Diabetes.  **4. Biochemical and Laboratory Data:** Fasting Blood Glucose, 1-hour and 2-hour Glucose during Oral Glucose Tolerance Test (OGTT), Glycated Hemoglobin (HbA1c), Lipid Profile (e.g., cholesterol, triglycerides), C-reactive Protein (CRP) levels, Insulin Levels and Insulin Resistance Indices (e.g., HOMA-IR).  **5. Vital Signs**: Blood Pressure, Heart Rate.  **6. Lifestyle Factors:** Diet (e.g., caloric intake, macronutrient composition), Physical Activity Level, Smoking Status, Alcohol Consumption.  **7. Imaging Data:** Fetal Measurements (e.g., abdominal circumference), Placental Thickness, Uterine Artery Doppler Velocities.  **8. Genetic and Epigenetic Data:** Single Nucleotide Polymorphisms (SNPs) linked to diabetes, Methylation Patterns in Candidate Genes, Transcriptomic or Proteomic Biomarkers.  **9. Data from Continuous Monitoring Devices**: Continuous Glucose Monitoring (CGM) Data, Activity Tracking Metrics (e.g., step count)  **10. Sociocultural and Environmental Factors**: Access to Healthcare, Education Level, Environmental Factors (e.g., air pollution exposure, urban vs. rural living), Insurance information.  **11. Medications**: Insulin. |
| 4.4 Specific Data input to AI algorithm | List specific data types such as: Age, BMI, Pregnancy weight, Blood pressure, comorbidities, etc. |
| **5. AI Models Characteristics** | |
| 5.1 Main AI models used | The main AI model architecture used, such as:   - Classical machine learning - Deep learning - Generative AI |
| 5.2 Specific AI models used. | Indicate the specific models or variants of AI architecture employed, such as:   - ML: Logistic regression (LR), random forests (RF), SVM, linear regression, Decision trees (DT), gradient boosting model (GB), Stochastic Gradient Descent (SGD), and extreme gradient boosting (XGBoost), Extreme randomized tree (ETC), AdaBoost Classifier (Adaptive Boosting, discrete AdaBoost), CatBoost Classifier (Categorical Boosting; CBC), Light Gradient Boosting Machine (LGBM), Gaussian Naïve Bayes (GNB) and Bernoulli Naïve Bayes (BNB), Extra Trees (ET), Balanced Random Forest (BRF). Transfer Learning (TL), Gradient Boosting (GB), AdaBoost, Histogram-based Gradient Boosting Classification Tree (HGB), Bagging algorithm, BAYESIAN NETWORK (BN), Bayesian Profile Hidden Markov Model (PHMM), generalized linear model (GLM), Classification and Regression Tree (CART). - DL: MLP (Multi-layer perceptron), Artificial Neural Network (ANN), CNN, RNN, Back propagation artificial neural network (BPNN), LSTM, radial basis function network (RBF Network). - LLM, transformers. |
| 5.3 Validation type | Specify whether the validation of the AI model was internal or external:   - **Internal Validation:** Refers to the validation performed within the same dataset used for training, often by splitting the dataset into subsets (e.g., training, validation, and testing sets). Common techniques include cross-validation (e.g., K-fold) and hold-out validation. - **External Validation:** Refers to the validation performed on an entirely separate dataset that was not used during the training process. This helps evaluate the model's generalizability to new, unseen data. - **Interna and External** |
| 5.4 Validation technique | The approach used to validate the DL algorithm: Hold-out cross-validation, K-fold cross-validation, Leave One Out cross-validation (LOOCV). |
| 5.5 Performance metrics | The measures used to evaluate the accuracy and effectiveness of the AI algorithm in the study, such as accuracy (ACC), AUC-ROC, PR-AUC, confusion matrix (CF), positive predictive value (PPV), F1-score, specificity (SPEC), and sensitivity (SENS), or other performance measures of the AI algorithm.  false positive rate (FPR), false negative rate (FNR), false discovery rate (FDR), false omission rate (FOR) and error rate, TP (true positive), TN (true negative), FP (false positive), FN (false negative). MSE (mean square error), RMSE |
| 5.6 Interpretability and Explainability | Shapley additives explanations (SHAP) - provides insight into how certain the model is about its predictions, Prediction Confidence (PC) - Confidence provides insights into uncertainty in model predictions, which is critical in high-stakes applications (e.g., healthcare), CONFIDEMCE INTERVAL (CI).  Others: PDPs, ICE plots, Feature Importance |
| 5.7 Training data (%) | The proportion of the dataset used for training the AI model, expressed as a percentage. For example, if the dataset is split into training and testing sets, indicate the percentage allocated to training (e.g., 70%, 80%, etc.). |
