# Supplementary material for: Artificial Intelligence in Gestational Diabetes Care: A Systematic Review
Source: J Diabetes Sci Technol. 2025 Aug 25:19322968251355967. Online ahead of print. doi: 10.1177/19322968251355967 (PMC12380749; doi:10.1177/19322968251355967)
Supplement: sj-docx-4-dst-10.1177_19322968251355967 – Supplemental material for Artificial Intelligence in Gestational Diabetes Care: A Systematic Review [file sj-docx-4-dst-10.1177_19322968251355967.docx]

**Multimedia Appendix 4: The modifed version of QUADAS-2**

**1. Participants (Patient Selection)**

| **Signaling Questions** | **Explanation** |
| --- | --- |
| **1.1 Was a consecutive or random sample of patients enrolled?** | - **Yes**: If a consecutive or random sample of eligible participants (pregnant women diagnosed or at risk of gestational diabetes) was enrolled.  - **No**: If patients were selected based on convenience or unclear selection criteria.  - **Unclear**: If the study did not report participant selection details. |
| **1.2 Did the study avoid inappropriate exclusions?** | - **Yes**: If inclusion/exclusion criteria were appropriate, ensuring a representative sample of gestational diabetes patients. - **No**: If participants were excluded in a way that could bias model performance (e.g., excluding high-risk groups, or including only those with well-controlled GDM).  - **Unclear**: If no details were provided. |
| **1.3 Was the sample size sufficient?** | - **Yes**: If at least 100 participants were included, or if studies with multiple samples per participant had at least 100 samples. - **No**: If fewer than 100 participants or samples were included. - **Unclear**: If the sample size was not clearly reported. |
| **1.4 Was there a balance in the number of patients across relevant subgroups?** | - **Yes**: If no subgroup comprised more than 66.7% of the sample (ensuring diverse representation of BMI, ethnicity, age, etc.).  - **No**: If any subgroup dominated the sample (>66.7%). - **Unclear**: If subgroup proportions were not reported. |
| **Risk of Bias Assessment: Could the selection of participants introduce bias?**   - **Low risk of bias**: If all signaling questions are **"Yes."** - **High risk of bias**: If any answer is **"No."** - **Unclear risk of bias**: If key information is missing. | |
| **Applicability Concerns: Do the participants match the review question?**   - **Low concern**: If the study population aligns with the target population of the review. - **High concern**: If the study includes a non-representative population (e.g., excluding high-risk pregnancies). - **Unclear concern**: If insufficient details are provided. | |

**2. Index Test (AI Models)**

| **Signaling Questions** | **Explanation** |
| --- | --- |
| **2.1 Were the AI models described in detail?** | - **Yes**: If the study provided technical details (e.g., model type, architecture, hyperparameters, features used). - **No**: If only the model name was reported, or key details were missing. - **Unclear**: If insufficient information was provided. |
| **2.2 Were all features (predictors) clearly identified?** | - **Yes**: If all features (clinical, biochemical, wearable data, etc.) were explicitly listed. - **No**: If feature selection was unclear or incomplete. - **Unclear**: If no details were provided. |
| **2.3 Were features assessed in the same way for all participants?** | - **Yes**: If all participants underwent standardized data collection. - **No**: If different methods/devices were used without standardization (e.g., multiple types of glucose monitors with inconsistent calibration). - **Unclear**: If assessment methods were not reported. |
| **2.4 Were features collected without knowledge of the outcome?** | - **Yes**: If AI predictors were selected independently of outcome data. - **No**: If outcome data influenced feature selection. - **Unclear**: If no details were provided. |
| **Risk of Bias Assessment: Could the index test introduce bias?**   - **Low risk of bias**: If all signaling questions are **"Yes."** - **High risk of bias**: If any answer is **"No."** - **Unclear risk of bias**: If key information is missing. | |
| **Applicability Concerns: Does the index test match the review question?**   - **Low concern**: If the AI model aligns with gestational diabetes prediction/diagnosis/treatment monitoring. - **High concern**: If the model is not directly relevant to the review question (e.g., developed for type 2 diabetes instead). - **Unclear concern**: If model applicability is not clear. | |

**3. Reference Standard (Ground Truth)**

| **Signaling Questions** | **Explanation** |
| --- | --- |
| **3.1 Was the reference standard likely to correctly classify the outcome (e.g., gestational diabetes diagnosis, risk prediction, treatment response)?** | - **Yes**: If clinical guidelines (e.g., ADA, WHO, IADPSG, OGTT criteria) or validated biomarkers were used. - **No**: If an unvalidated reference test was used. - **Unclear**: If the reference standard was not well-defined. |
| **3.2 Was the outcome determined in a consistent manner for all participants?** | - **Yes**: If the same diagnostic criteria were applied to all participants. - **No**: If different criteria were applied inconsistently. - **Unclear**: If criteria were not reported. |
| **3.3 Was the outcome determined without knowledge of predictor data?** | - **Yes**: If outcome classification was blinded to AI model predictions. - **No**: If assessors had access to AI-generated predictions when classifying outcomes. - **Unclear**: If blinding was not reported. |
| **3.4 Was there a time interval between predictor assessment and outcome determination that could introduce bias?** | **- Yes:** If the outcome (e.g., gestational diabetes diagnosis, risk prediction, or treatment response) was determined within a reasonable timeframe after predictor data collection, minimizing the risk of changes in patient status.  **- No:** If there was a long or inappropriate time gap between predictor data collection and outcome determination, leading to potential misclassification or changes in patient condition.  **- Unclear:** If the study did not report the time interval between predictor assessment and outcome determination. |
| **Risk of Bias Assessment: Could the reference standard introduce bias?**   - **Low risk of bias**: If all signaling questions are **"Yes."** - **High risk of bias**: If any answer is **"No."** - **Unclear risk of bias**: If key information is missing. | |
| **Applicability Concerns: Does the reference standard match the review question?**   - **Low concern**: If the reference standard is appropriate for gestational diabetes. - **High concern**: If the reference standard is inappropriate (e.g., inconsistent diagnostic thresholds). - **Unclear concern**: If reference standard details are unclear. | |

**4. Analysis**

| **Signaling Questions** | **Explanation** |
| --- | --- |
| **4.1 Were all participants included in the analysis?** | - **Yes**: If no participants were inappropriately excluded. - **No**: If participants were excluded without justification. - **Unclear**: If inclusion/exclusion criteria for analysis were unclear. |
| **4.2 Was data preprocessing carried out appropriately?** | - **Yes**: If missing data was handled appropriately (e.g., imputation). - **No**: If data preprocessing methods were flawed or not reported. - **Unclear**: If no details were provided. |
| **4.3 Was the breakdown of training, validation, and test sets appropriate?** | - **Yes**: If data was split using best practices (e.g., 70-80% training, 10-15% validation, 10-20% test). - **No**: If an inappropriate split was used. - **Unclear**: If data splitting details were missing. |
| **4.4 Was the performance of the model evaluated appropriately?** | **- Yes:** If the confusion matrix was presented,  Or more than one measure was used and the selected measures were appropriate.  **- No:** If the confusion matrix was not presented, and only one measure was reported,  Or the selected measures were not appropriate.  **- Unclear:** If no information was provided on the performance measures |
| **Risk of Bias Assessment: Could the analysis introduce bias?**   - **Low risk of bias**: If all signaling questions are **"Yes."** - **High risk of bias**: If any answer is **"No."** - **Unclear risk of bias**: If key information is missing. | |
