# Supplementary material for: Artificial Intelligence in Gestational Diabetes Care: A Systematic Review
Source: J Diabetes Sci Technol. 2025 Aug 25:19322968251355967. Online ahead of print. doi: 10.1177/19322968251355967 (PMC12380749; doi:10.1177/19322968251355967)

**Multimedia Appendix 5: Reviewers’ judgments about each domain in “risk of bias” and "applicability concerns" for each included study**


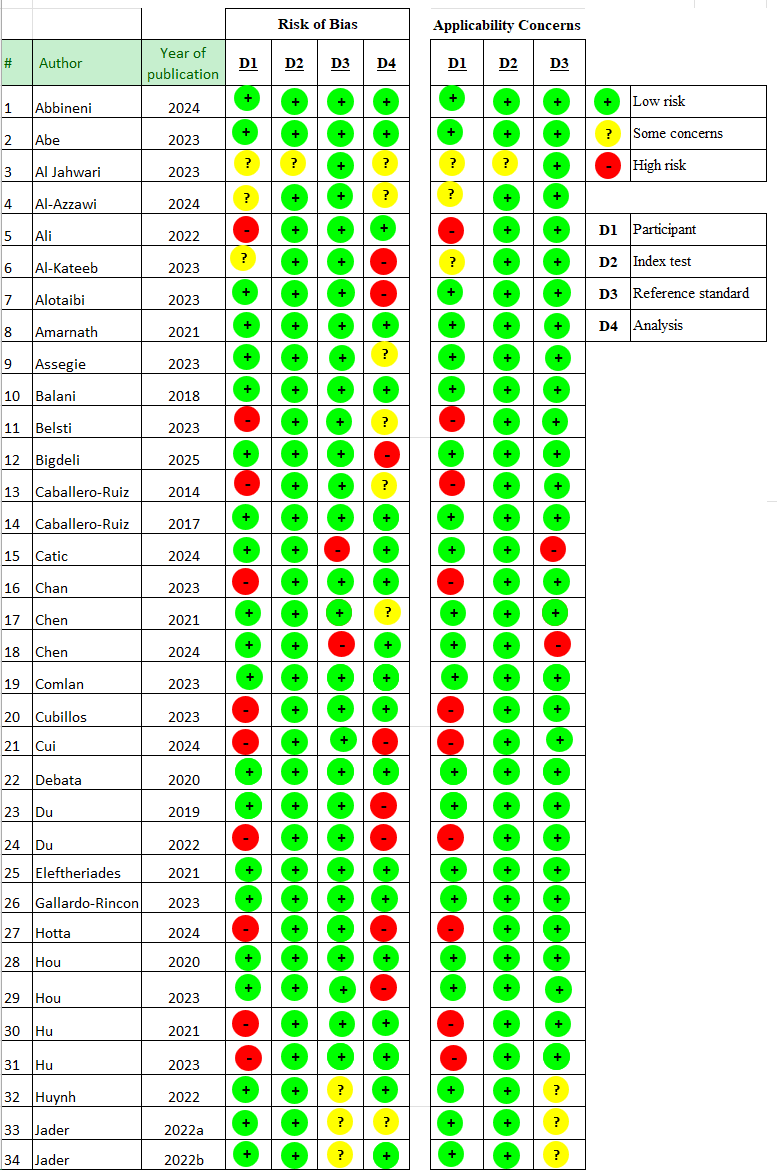


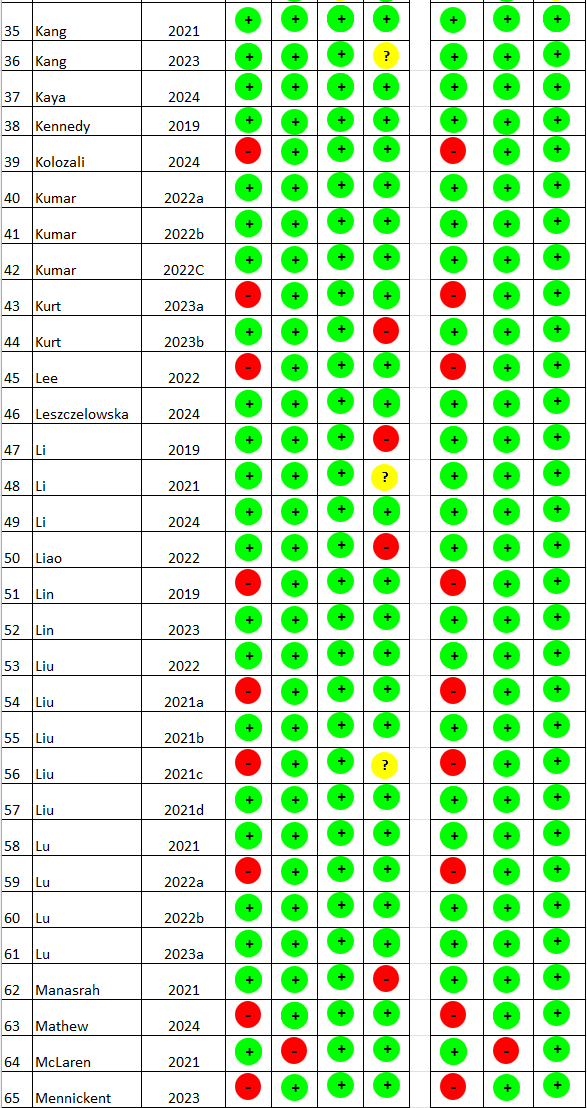


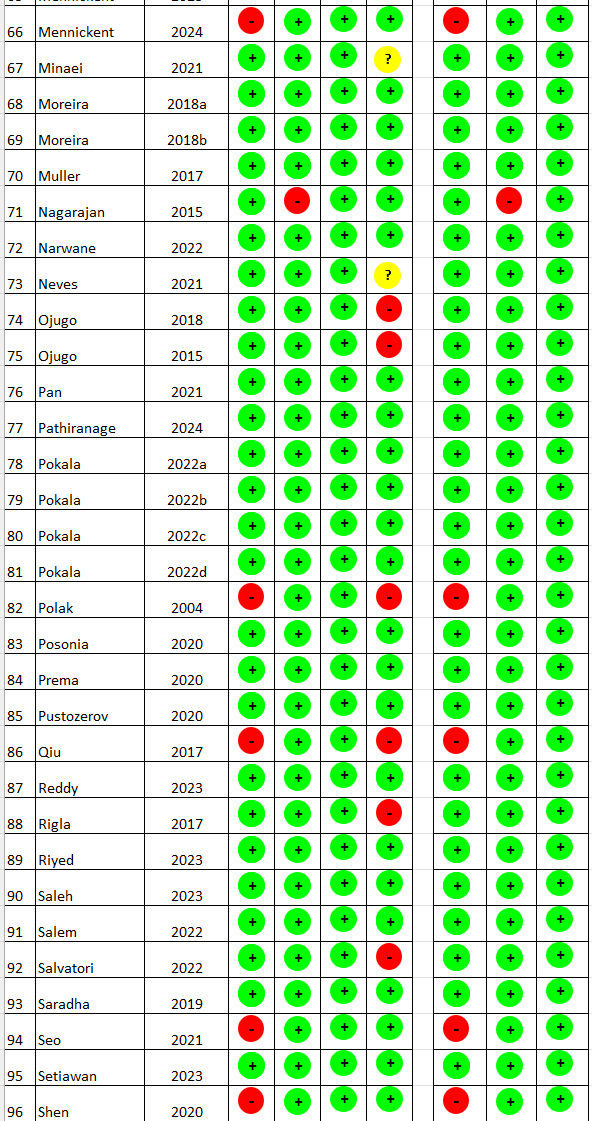


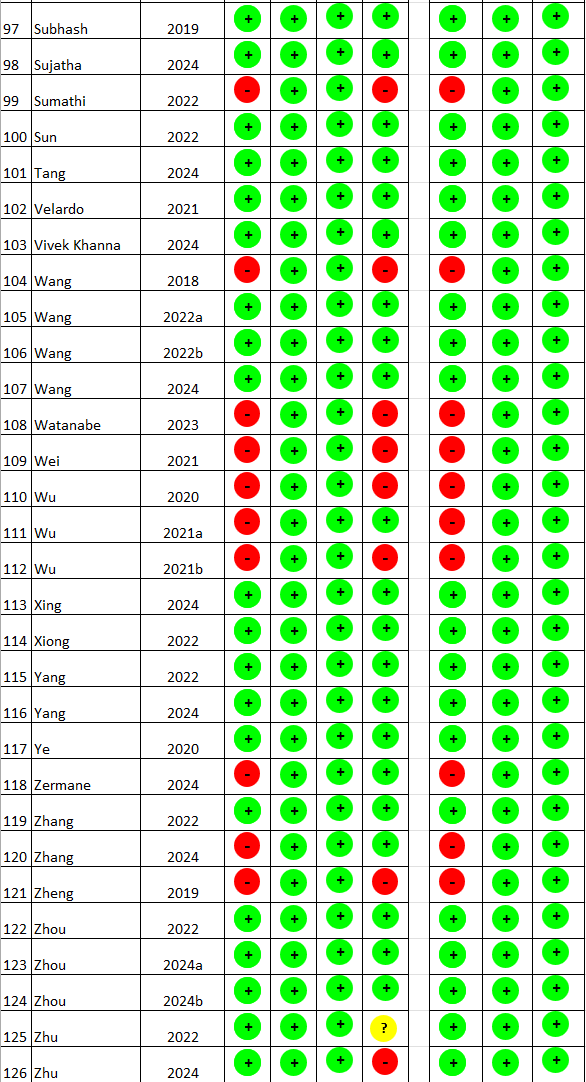

Supplement: sj-docx-5-dst-10.1177_19322968251355967 – Supplemental material for Artificial Intelligence in Gestational Diabetes Care: A Systematic Review [file sj-docx-5-dst-10.1177_19322968251355967.docx]
